# Effects of spatial distance and woody plant cover on beta diversity point to dispersal limitation as a driver of community assembly during postfire succession in a Mediterranean shrubland

**DOI:** 10.1002/ece3.9130

**Published:** 2022-07-24

**Authors:** Iván Torres, Antonio Parra, José M. Moreno

**Affiliations:** ^1^ Departamento de Ciencias Ambientales Universidad de Castilla‐La Mancha Toledo Spain

**Keywords:** community assembly, distance decay of similarity, nestedness, niche processes, postfire dynamics, turnover

## Abstract

Beta diversity, and its components of turnover and nestedness, reflects the processes governing community assembly, such as dispersal limitation or biotic interactions, but it is unclear how they operate at the local scale and how their role changes along postfire succession. Here, we analyzed the patterns of beta diversity and its components in a herbaceous plant community after fire, and in relation to dispersal ability, in Central Spain. We calculated multiple‐site beta diversity (β_SOR_) and its components of turnover (β_SIM_) and nestedness (β_SNE_) of all herbaceous plants, or grouped by dispersal syndrome (autochory, anemochory, and zoochory), during the first 3 years after wildfire. We evaluated the relationship between pairwise beta diversity (β_sor_), and its components (β_sim_, β_sne_), and spatial distance or differences in woody plant cover, a proxy of biotic interactions. We found high multiple‐site beta diversity dominated by the turnover component. Community dissimilarity increased with spatial distance, driven mostly by the turnover component. Species with less dispersal ability (i.e., autochory) showed a stronger spatial pattern of dissimilarity. Biotic interactions with woody plants contributed less to community dissimilarity, which tended to occur through the nestedness component. These results suggest that dispersal limitation prevails over biotic interactions with woody plants as a driver of local community assembly, even for species with high dispersal ability. These results contribute to our understanding of postfire community assembly and vegetation dynamics.

## Graphical abstract

In this work we analyze how beta diversity, and its components of turnover and nestedness, change in a herbaceous plant community during the first years following fire, and how they relate to spatial distance and the effect of woody plant cover. We found that dispersal limitation, indicated by an increase in similarity with spatial distance, is the main emergent pattern, while the role of woody plant cover, as a proxy of biotic interactions, contributes much less to community dissimilarity. Additionally, we found that turnover is the dominant component of beta diversity.

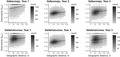

## 1 INTRODUCTION

Beta diversity, or the variation of species composition across samples, is a fundamental characteristic of biological communities that manifests itself throughout all scales of life on Earth (Anderson et al., 2011; Soininen, Lennon, & Hillebrand, 2007a; Tuomisto, 2010; Whittaker, 1960). It is the result of the processes that assemble biological communities, and therefore, beta diversity patterns may inform about the processes that govern community assembly and function (Baselga, 2010; Mori et al., 2018; Myers et al., 2015; Siefert et al., 2013), help testing ecological theories (Baselga, 2010) and inform conservation priorities and planning (Angeler, 2013; Socolar et al., 2016). Beta diversity can be partitioned into two components: turnover, or species replacement, and nestedness, or differences in composition because sites with fewer species have biotas that are subsets of sites with more species (Baselga, 2010; Soininen et al., 2018). These components can provide further insights into community assembly (Dobrovolski et al., 2012; Gutiérrez‐Cánovas et al., 2013; Mori et al., 2018; Svenning et al., 2011).

Biological communities are assembled through dispersal limitation, environmental heterogeneity, and biotic interactions. In addition, stochastic variation can contribute to beta diversity patterns (Chase & Myers, 2011; Tuomisto et al., 2003; Ulrich et al., 2016). These drivers are not mutually exclusive and can operate at different spatial scales (Chase & Myers, 2011; Keil et al., 2012; Laliberté et al., 2009; Qian et al., 2005). First, dispersal limitation promotes variation in community composition, creating a pattern of increasing dissimilarity with increasing distance between samples, known as distance decay of similarity, which is a universal trend in ecology at a wide range of spatial scales (Keil et al., 2012; Soininen, Mcdonald, & Hillebrand, 2007b). The pattern of distance decay is linked to the mobility of organisms, and groups of organisms with different dispersal abilities show different values of beta diversity or its components (Dobrovolski et al., 2012; Si et al., 2015; Soininen et al., 2018). Second, environmental heterogeneity, related to abiotic factors such as climate, topography, or geological substrate at global or regional scales, causes habitat filtering through niche‐based processes (Diamond, 1975; Jankowski et al., 2009; Kraft & Ackerly, 2014), while at local scales microtopography, soil, and other microenvironmental factors can affect community assembly (Lundholm, 2009). Third, biotic interactions (e.g., competition, herbivory, or facilitation) also contribute a large fraction to community variation (Diamond, 1975; García‐Girón et al., 2020; Kraft & Ackerly, 2014; Poisot et al., 2015). Moreover, biotic interactions, particularly non‐trophic ones (Kéfi et al., 2012), cause microenvironmental change, such as in ecological succession, where the increase in aboveground biomass, leaf litter, and partitioning of resources create microhabitat heterogeneity that drive compositional change (Kouba et al., 2014; Sabatini et al., 2014; White & Jentsch, 2004).

Disturbances are on themselves an ecological filter (White & Jentsch, 2004), and fire acts as a primary selective agent that deterministically influences community composition (Harms et al., 2017; Myers & Harms, 2011). Fire is a key driver of biodiversity in fire‐prone regions of the world (He et al., 2019), with burned areas supporting high levels of community heterogeneity (beta diversity) (Guo, 2001; Schwilk et al., 1997). In Mediterranean‐type environments, the release of resources and reduced competition with woody plants caused by fire promotes an increase in herbaceous species richness (alpha diversity) during the first or second postfire year, and a decrease shortly thereafter as woody plants develop and canopy cover increases (Barro & Conard, 1991; Calvo et al., 2005; Capitanio & Carcaillet, 2008; Keeley et al., 2005, 2012; Parra & Moreno, 2018). Therefore, postfire environments provide an opportunity for exploring community assembly patterns (Han et al., 2018; Harms et al., 2017; Myers et al., 2015; White & Jentsch, 2004). Along postfire succession, dispersal, species interactions, and stochastic processes can affect community assembly (Han et al., 2018; Harms et al., 2017; Måren et al., 2018), but how these processes affect the turnover and nestedness components of beta diversity needs to be further explored (but see Heydari et al. (2017), Han et al. (2018)). From an applied point of view, understanding how biodiversity changes after fire in fire‐prone ecosystems helps us understand the processes that shape these communities and may help us address relevant management implications to preserve biodiversity (Foley et al., 2005; Kelly & Brotons, 2017).

Here, we analyzed the patterns of beta diversity and its components in the herbaceous plant community of a Mediterranean shrubland during the first 3 years after fire and in an adjacent unburned stand, with a focus on spatial patterns of dissimilarity and biotic interactions with woody plants, for groups of species with different dispersal ability. Our questions were as follows: (1) How does beta diversity of the herbaceous plant community and its components of turnover and nestedness change with time since fire? (2) How does beta diversity and its components relate to spatial distance and to the influence of woody species? (3) Do these beta diversity patterns differ among groups of species with different dispersal modes?

## 2 MATERIALS AND METHODS

### 2.1 Study area

This study was carried out after a large summer wildfire occurred in August 1st, 2002, in Central Spain (Anchuras, province of Ciudad Real; 587 m a.s.l.; 39°27′N, 4°52′W). The fire burned ca. 1500 ha of different vegetation types, including shrublands, oak and pine woodlands, and crops distributed over a landscape known as “Raña,” that is, alluvial flatlands crossed by ravines. We focused on a Mediterranean abandoned *dehesa* of sparse *Quercus suber* L. trees and other *Quercus* species, with a prefire estimated cover around 15%, that had been encroached by shrubs (mainly *Cistus ladanifer* L., *Rosmarinus officinalis* L., *Phillyrea angustifolia* L. and *Erica* spp.). No record of previous fires at the site is available, but these were unlikely given the fuel structure before abandonment, according to aerial images from 1956, and given the recent history of forest fires in Spain, which shows that wildfires were not so common prior to the last decades of the 20th century (Moreno et al., 1998). Climate is Mediterranean, with an average total annual rainfall of 544 mm, mean minimum temperature of 7.4°C, and mean maximum temperature of 20.3°C (Embalse de Torre de Abraham meteorological station; AEMET, Spain). The substrate is alluvial, and the soils are mainly Entisols (CNIG, 2006).

### 2.2 Sampling design

**FIGURE 1:**
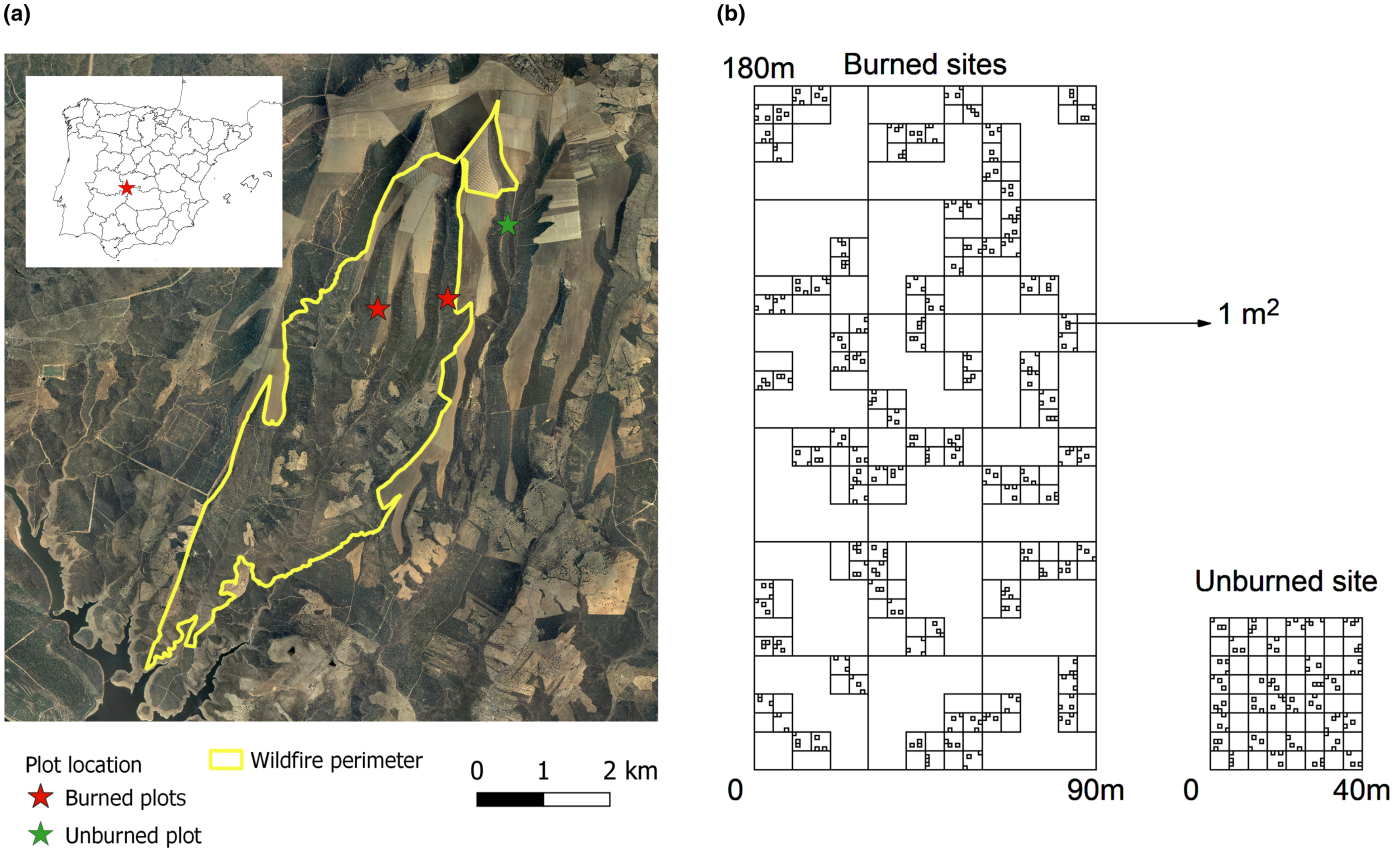
(a) Location of study site—red star—in Central Spain. (b) Location of sampled plots and fire perimeter. Aerial image from FotoPNOA 2004 to 2021 CC‐BY 4.0 scne.Es. (c) Schematic representation of the sampling layout at the burned sites (Valbermejo and Valdehalcones) and the adjacent unburned site. At the burned sites, 324 1 m^2^ squares were sampled from a grid of nested, randomly chosen 5 × 5, 10 × 10 and 30 × 30 m squares. At the unburned site, 94 1 m^2^ squares were sampled from a 5 × 5 m grid (see text for details).

Within the burned area, we selected two adjacent valleys in west‐facing slopes: Valbermejo and Valdehalcones, with 33% and 36% slope, respectively, where a multiscale nested sampling was implemented. At each valley, we established one permanent sampling plot, 90 × 180 m in size, and we divided it in nested grids of 30, 10, 5, and 1 m. Three 10 m grid cells were randomly selected within each 30 m cell, and within each selected 10 m cell, two 5 m cells were selected. Finally, three cells of the 1 m grid were randomly selected within each 5 m cell. This hierarchical design resulted in 324 sampling quadrats of 1x1 m in each plot that were spread along a wide range of distances (Figure 1, also Viedma et al., 2012). Field sampling was carried out in June and July—after all species had flowered and/or set fruit—of the first, second, and third years after fire (from here on Year 1, Year 2, and Year 3). To explore diversity patterns in unburned vegetation, in 2006, an additional 40 × 40 m plot was selected in an unburned site adjacent to the fire perimeter, and 94 1 × 1 m quadrats were established as described in Torres et al. (2013) (Figure 1). In each of the sampling quadrats, we recorded the presence/absence of all vascular species and visually estimated the cover of herbaceous plants, shrubs, and trees. Most trees were resprouting from ground level except for *Q. suber* that resprouted both from the ground or from the aerial buds. Tree and shrub cover were used to calculate the percentage of ground covered by these woody species. We used woody plant cover as a proxy for biotic interactions because woody plants locally affect the access to light, water, nutrients, and space, and are one of the main drivers of herbaceous species diversity in Mediterranean environments. This is supported by the close relationships between herbaceous cover and richness and woody plant cover during the first few years after fire in similar shrubland ecosystems (Céspedes et al., 2014; Parra & Moreno, 2018). Several plots could not be resampled in the campaigns of the second or third year because they were impossible to find or had signs of having been altered; thus, the number of plots used in the analyses that involved all years was 312 and 308 in Valbermejo and Valdehalcones, respectively.

### 2.3 Seed dispersal syndromes

Herbaceous species were assigned to one of the following dispersal modes: autochory (seeds or propagules either with self‐propagation mechanisms or with no morphological adaptation to dispersal—i.e., barochory‐), anemochory (the presence of wings or pappus), and zoochory (including epizoochory, endozoochory, and myrmecochory). Dispersal mode was assigned following the criteria by McIntyre et al. (1995) and Perez‐Harguindeguy et al. (2016) based on seed or propagule morphology, according to the descriptions and illustrations in Valdés et al. (1987), Blanca et al. (2009) and Castroviejo (1986–2012). The species list and dispersal mode can be found in Appendix S1.

### 2.4 Statistical analyses

Our main goal was to detect year‐to‐year changes in herbaceous species beta diversity, its spatial patterns, and the effect of biotic interactions with woody plants. Hence, we used the presence/absence data of only the herbaceous species in the 1 m^2^ quadrats. Differences in herbaceous species richness and in woody and herbaceous plant cover were tested with generalized linear mixed models for each site with Year as fixed effect. For species richness, we used the function glmer from the package lme4 with a Poisson distribution, and included sampling quadrat ID nested within 10 m plot ID as a random effect to account for repeated measures and for short‐distance spatial autocorrelation. Visual inspection of semivariograms showed that most spatial autocorrelation was under ca. 10 m. For woody and herbaceous plant cover, we used the function *glmmTMB* from package *glmmTMB* with a beta distribution with zero inflation, and with sampling quadrat included as a random effect. Significance of main effects was assessed with the function ANOVA from package *car*, and differences among years were tested with a Tukey test with the function *glht* from the package *multcomp* in R version 4.1.3 (R core team, 2022).

We quantified overall beta diversity at each site, over each of the first 3 years after fire, or in the unburned control, for all herbaceous species and for groups of species with different dispersal modes, as multiple‐site dissimilarities (i.e., one measure per site and year) using the multiple‐site Sorensen dissimilarity (β_SOR_) from R package *betapart*. This was then decomposed into its two components, turnover (β_SIM_) and nestedness (β_SNE_) (Baselga, 2010). We also calculated the β_ratio_ as the ratio between β_SNE_ and β_SOR_, where values smaller than 0.5 indicate that turnover is the dominant component of beta diversity. To explore the pattern of beta diversity in space and the effect of biotic interactions with woody plants, we first calculated pairwise beta diversity metrics between all possible pairs of sampling quadrats as pairwise‐site dissimilarity (β_sor_), and its turnover (β_sim_) and nestedness (β_sne_) components (Baselga, 2010). Spatial (geographic) distance among sampling quadrats was calculated as Euclidian distance, and the difference in woody plant cover (%) between sampling quadrats was calculated.

The relationship between differences in woody plant cover and pairwise beta diversity and its components was analyzed with multiple regression on distance matrices (MRM) (Lichstein, 2007). As woody plant cover is likely to be spatially autocorrelated, thus possibly inflating the significance of the test, we assessed the significance of the relationship with a partial mantel tests of each beta diversity matrix on the difference in woody plant cover matrix while controlling for the spatial distance matrix, assessing its significance with 9999 permutations. The spatial pattern of beta diversity was analyzed with a mantel test with 9999 permutations. Finally, we calculated the slope and intercept coefficients of MRM. All analyses were performed with R version 4.1.3 (R core team, 2022), using the packages *nlme*, *multcomp*, *betapart,* and *vegan*, as well as *ggplot2* for graphs.

## 3 RESULTS

### 3.1 Species richness and plant cover

**FIGURE 2:**
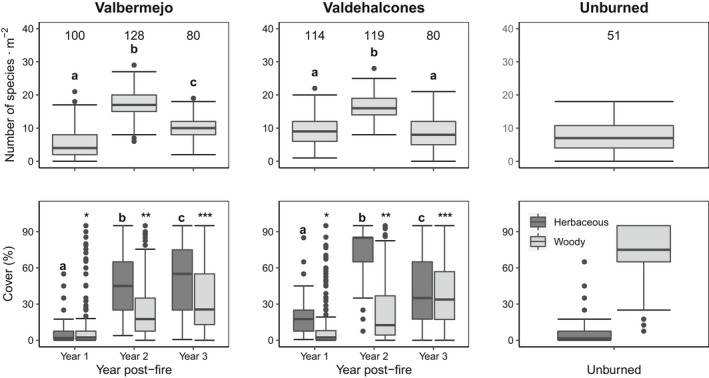
Species richness of herbaceous plants (top) and cover of herbaceous and woody plants (bottom) in the first 3 years after a fire in a Mediterranean shrubland (two sites, Valbermejo and Valdehalcones) and in an adjacent unburned stand. Numbers above boxplots in top row indicate regional species pool (gamma diversity). Letters and symbols indicate significant differences at level *p* < .05 after a Tukey test.

Herbaceous species richness significantly changed over the years (X^2^ = 316.2, *p* < .001 for Valbermejo; X^2^ = 191.0, *p* < .001 for Valdehalcones). It significantly increased from Year 1 to Year 2, and then decreased in Year 3 (Figure 2). Herbaceous cover significantly changed across years at both sites (X^2^ = 1138.8, *p* < .001 for Valbermejo; X^2^ = 1017.5, *p* < .001 for Valdehalcones), with a marked increase in Year 2 and a decrease in Year 3 at Valdehalcones (Figure 2). Woody plant cover significantly increased over time at both sites (X^2^ = 1087.3, *p* < .001 for Valbermejo; X^2^ = 1139.4, *p* < .001 for Valdehalcones, Figure 2). Species richness at Year 3 at the burned sites resembled that of the unburned site (not tested), which had a herbaceous cover much lower and a woody cover much higher than the burned ones (Figure 2).

### 3.2 Multiple‐site beta diversity

Multiple‐site beta diversity was high for all sites, years, and groups of species. When all species were considered together, beta diversity decreased from Year 1 to Year 2, and increased in Year 3. At the unburned site, beta diversity was also high, even if lower than at the burned sites (Table 1). The main component of beta diversity was turnover, with nestedness being extremely low. Nestedness decreased from Year 1 to Year 2, and increased in Year 3 at both sites. The unburned site had a nestedness component one order of magnitude higher than the burned ones (Table 1).

**TABLE 1:** Multiple‐site Sørensen dissimilarity (β_SOR_), its components of turnover (β_SIM_) and nestedness (β_SNE_) and the β_ratio_ (the ratio between β_SNE_ and β_SOR_) of herbaceous plants with different dispersal syndromes in the first 3 years after a fire in a Mediterranean shrubland (two sites, Valbermejo and Valdehalcones) and in an adjacent unburnt stand

	Dissimilarity (βSOR)	Turnover (βSIM)	Nestedness (βSNE)	β _ratio_
All species				
Valbermejo				
Year 1	0.992	0.985	0.007	0.007
Year 2	0.988	0.985	0.003	0.003
Year 3	0.989	0.985	0.004	0.005
Valdehalcones				
Year 1	0.991	0.986	0.005	0.005
Year 2	0.989	0.986	0.003	0.003
Year 3	0.990	0.981	0.008	0.009
Unburned	0.965	0.929	0.036	0.038
Autochory				
Valbermejo				
Year 1	0.991	0.984	0.007	0.008
Year 2	0.992	0.989	0.003	0.003
Year 3	0.991	0.986	0.005	0.005
Valdehalcones				
Year 1	0.992	0.986	0.005	0.005
Year 2	0.992	0.989	0.003	0.003
Year 3	0.990	0.982	0.008	0.008
Unburned	0.960	0.936	0.024	0.025
Anemochory				
Valbermejo				
Year 1	0.988	0.979	0.009	0.009
Year 2	0.982	0.969	0.013	0.013
Year 3	0.986	0.971	0.014	0.015
Valdehalcones				
Year 1	0.990	0.983	0.007	0.007
Year 2	0.983	0.972	0.011	0.012
Year 3	0.988	0.971	0.017	0.017
Unburned	0.958	0.908	0.051	0.053
Zoochory				
Valbermejo				
Year 1	0.989	0.981	0.008	0.008
Year 2	0.987	0.979	0.008	0.009
Year 3	0.989	0.981	0.008	0.008
Valdehalcones				
Year 1	0.988	0.975	0.013	0.013
Year 2	0.988	0.980	0.009	0.009
Year 3	0.989	0.979	0.010	0.010
Unburned	0.952	0.897	0.055	0.058

When considering dispersal modes, species with anemochory had the lowest beta diversity values in most cases, while species with autochory showed the highest values (Table 1). Most of the beta diversity was attributable to the turnover component, nestedness being extremely low. Species with autochory had the highest turnover and lowest nestedness compared with the other dispersal modes (Table 1).

### 3.3 Spatial patterns of beta diversity

**FIGURE 3:**
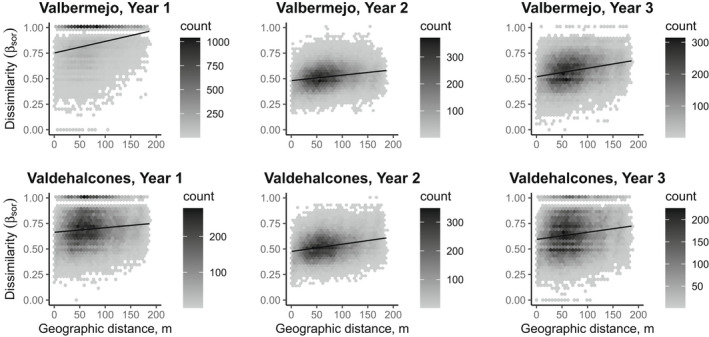
Relationships of pairwise beta diversity (β_sor_) and geographic distance for herbaceous plants in the first 3 years after a fire in a Mediterranean shrubland (two sites, Valbermejo and Valdehalcones). Lines indicate significant multiple regression models assessed with mantel tests with 9999 permutations.

At the burned sites, and for all species, pairwise beta diversity significantly increased with spatial distance regardless of time since fire (Table 2, Figure 3). The slope of the relationship (i.e., the strength of the decay in similarity), however, was different between burned sites: At Valbermejo, it was maximum in Year 1, decreased by half in Year 2, and increased in Year 3. At Valdehalcones, it started with a low value, increased to a value similar to that of Valbermejo, and remained around that value in Year 3 (Table 2, Figure 3). The slope of pairwise beta diversity vs spatial distance was higher at the unburned site (Table 2). The intercept of the regression dropped markedly from Year 1 to Year 2 and increased to an intermediate value in Year 3. At the unburned site, the intercept showed an intermediate value of 0.55 (Table 2).

**TABLE 2:** Coefficients of multiple regression models between differences in spatial distance or in woody plant cover, and pairwise beta diversity (β_sor_) and its components of turnover (β_sim_) and nestedness (β_sne_) of herbaceous plants with different dispersal syndromes in the first 3 years after a fire in a Mediterranean shrubland (two sites, Valbermejo and Valdehalcones) and in an adjacent unburned stand

	Difference in spatial distance						Difference in woody plant cover					
	Dissimilarity (β_sor_)		Turnover (β_sim_)		Nestedness (β_sne_)		Dissimilarity (β_sor_)		Turnover (β_sim_)		Nestedness (β_sne_)	
	Intercept	Slope	Intercept	Slope	Intercept	Slope	Intercept	Slope	Intercept	Slope	Intercept	Slope
All species												
Valbermejo												
Year 1	**0.75**	**0.00114**	**0.62**	**0.00126**	0.13	−0.00012	0.83	0.00025	0.72	−0.00037	**0.11**	**0.00063**
Year 2	**0.48**	**0.00053**	**0.40**	**0.00065**	0.08	−0.00011	0.51	0.00018	0.44	0.00018	0.07	0.00001
Year 3	**0.52**	**0.00083**	**0.41**	**0.00094**	0.11	−0.00010	**0.57**	**0.00044**	0.47	0.00028	0.09	0.00016
Valdehalcones												
Year 1	**0.66**	**0.00045**	**0.53**	**0.00056**	0.13	−0.00011	**0.69**	**0.00041**	0.57	0.000127	**0.12**	**0.00028**
Year 2	**0.48**	**0.00071**	**0.42**	**0.00070**	0.06	0.00001	**0.51**	**0.00045**	**0.46**	**0.00038**	0.06	0.00007
Year 3	**0.59**	**0.00070**	**0.42**	**0.00067**	0.17	0.00003	**0.60**	**0.00141**	0.46	0.00017	**0.13**	**0.00124**
Unburned	**0.55**	**0.00425**	**0.35**	**0.00354**	0.19	0.00071	0.66	−0.00071	0.49	−0.00200	**0.17**	**0.00129**
Autochory												
Valbermejo												
Year 1	**0.72**	**0.00142**	**0.59**	**0.00166**	0.13	−0.00024	0.83	−0.00026	0.72	−0.00052	0.10849	0.00025
Year 2	**0.68**	**0.00084**	**0.59**	**0.00109**	0.10	−0.00024	**0.74**	**0.00036**	0.66	0.00029	0.07748	0.00007
Year 3	**0.66**	**0.00131**	**0.50**	**0.00196**	0.16	−0.00064	0.75	0.00020	0.63	0.00036	0.11243	−0.00016
Valdehalcones												
Year 1	**0.70**	**0.00069**	**0.57**	**0.00088**	0.13	−0.00019	0.75	0.00022	0.64	0.000211	0.11392	0.00001
Year 2	**0.69**	**0.00071**	**0.61**	**0.00084**	0.08	−0.00013	0.74	0.00026	0.67	0.00030	0.07194	−0.00004
Year 3	**0.63**	**0.00127**	**0.46**	**0.00169**	0.17	−0.00042	0.71	0.00034	0.58	0.00018	0.13299	0.00016
Unburned	**0.68**	**0.00416**	**0.55**	**0.00503**	0.13	−0.00087	0.75	0.00044	0.63	0.00064	0.12124	−0.00020
Anemochory												
Valbermejo												
Year 1	**0.69**	**0.00111**	**0.54**	**0.00155**	0.15	−0.00043	0.77	0.00002	0.65	−0.00002	0.11554	0.00004
Year 2	0.33	0.00013	0.21	0.00010	0.12	0.00002	**0.32**	**0.00053**	0.21	0.00032	0.11452	0.00021
Year 3	**0.40**	**0.00068**	**0.22**	**0.00070**	0.17	−0.00001	**0.42**	**0.00103**	**0.25**	**0.00071**	**0.16169**	**0.00032**
Valdehalcones												
Year 1	0.70	0.00015	0.56	0.00018	0.14	−0.00002	0.71	0.00003	0.58	−0.00001	0.137931	0.00004
Year 2	**0.32**	**0.00061**	**0.22**	**0.00045**	**0.10**	**0.00015**	**0.35**	**0.00066**	**0.23**	**0.00053**	0.11162	0.00013
Year 3	**0.51**	**0.00044**	**0.28**	**0.00037**	0.23	0.00007	**0.49**	**0.00140**	0.31	−0.00034	**0.18093**	**0.00174**
Unburned	**0.51**	**0.00309**	**0.30**	**0.00294**	0.21	0.00014	0.59	−0.00075	0.41	−0.00197	**0.17992**	**0.00122**
Zoochory												
Valbermejo												
Year 1	**0.61**	**0.00157**	**0.45**	**0.00202**	0.16	−0.00045	0.73	−0.00042	0.61	−0.00137	**0.11707**	**0.00095**
Year 2	**0.42**	**0.00078**	**0.27**	**0.00089**	0.15	−0.00010	0.47	0.00008	0.33	0.00030	0.14736	−0.00021
Year 3	**0.51**	**0.00067**	**0.33**	**0.00073**	0.17	−0.00005	0.55	0.00017	0.39	0.00001	0.16580	0.00016
Valdehalcones												
Year 1	**0.49**	**0.00035**	0.29	0.00024	0.20	0.00011	0.51	0.00042	0.30	0.00034	0.20245	0.00009
Year 2	**0.47**	**0.00067**	**0.31**	**0.00064**	0.16	0.00003	0.51	0.00020	0.35	0.00015	0.16372	0.00004
Year 3	**0.60**	**0.00065**	**0.42**	**0.00062**	0.18	0.00002	**0.61**	**0.00107**	**0.42**	**0.00121**	0.19067	−0.00014
Unburned	**0.38**	**0.00565**	**0.16**	**0.00606**	0.22	−0.00041	0.53	−0.00099	0.33	−0.00127	0.20743	0.00028

*Note*: Bold indicates significant (*p* < .05) relationships, assessed with mantel test (differences in spatial distance) or with partial mantel test controlling for spatial distance (differences in woody cover) with 9999 permutations.

The turnover component of beta diversity showed a significant relationship with sampling distance in all cases. In contrast, the nestedness component showed no spatial pattern at both sites and all 3 years after fire. The intercept of the regression for both the turnover and the nestedness components showed a pattern that in most cases resembled that of pairwise beta diversity (Table 2).

The groups of species with different dispersal syndromes showed differences in the regression between beta diversity and spatial distance. In species with low‐to‐moderate dispersal ability (autochory and zoochory), a significant spatial pattern of beta diversity was found in all cases. The slope of the regression changed along time like the full set of species, but with different intensities. Only for species with anemochory, there was a lack of spatial pattern in Years 1 and 2 at the Valdehalcones and Valbermejo sites, respectively (Table 2). At the unburned site, there was also a spatial pattern of beta diversity, and it was higher in species with zoochory and lower in species with anemochory (Table 2). The intercept of the regression behaved differently among dispersal types, and the drop in similarity at distance 0 from Year 1 to Year 2 was much sharper for species with high dispersal ability (anemochory) than for those with low dispersal (autochory) (Table 2). At the unburned site, the intercept was higher for species with autochory than for species with anemochory and zoochory (Table 2).

The turnover component of beta diversity significantly increased with distance at all sites and times since fire for species with autochory, and the relationship was stronger than in the other groups of species. In the case of species with zoochory, all relationships were significant except for the Valdehalcones site in Year 1. For species with anemochory, the relationship was significant in all cases except at the Valdehalcones and Valbermejo sites in Years 1 and 2, respectively. The nestedness component of beta diversity only showed a significant relationship with distance in the case of species with anemochory in Year 2 at the Valdehalcones site (Table 2).

### 3.4 Effects of woody cover on beta diversity

**FIGURE 4:**
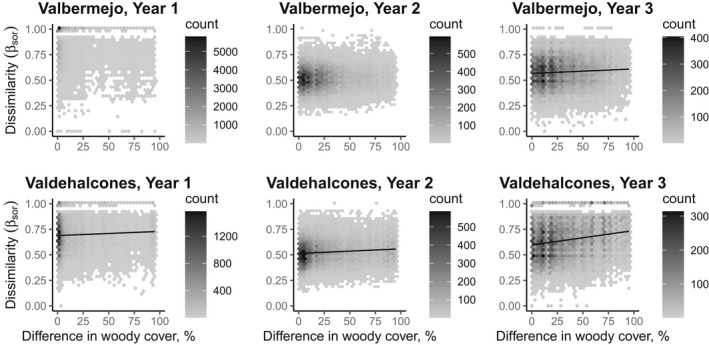
Relationships of pairwise beta diversity (β_sor_) and differences in woody plant cover for herbaceous plants in the first 3 years after a fire in a Mediterranean shrubland (two sites, Valbermejo and Valdehalcones). Lines indicate significant multiple regression models assessed with partial mantel test controlling for spatial distance with 9999 permutations.

Pairwise beta diversity showed a significant relationship with difference in woody plant cover at the Valbermejo site in Year 3 and at the Valdehalcones site in all postfire years (Table 2, Figure 4). No significant relationship was found between woody plant cover and beta diversity at the unburned site (Table 2). The intercept of the regression (i.e., the dissimilarity between quadrats with similar woody cover) decreased strongly from Year 1 to Year 2 and increased to an intermediate value in Year 3 (Table 2, Figure 4). For the turnover component of beta diversity, the relationship with differences in woody plant cover was significant only in Year 2 at the Valdehalcones site, while the relationship with the nestedness component was significant in Year 1 at both sites and at the Valdehalcones site in Year 3, as well as at the unburned site (Table 2).

The relationship between beta diversity and the difference in woody plant cover differed across dispersal modes. For species with anemochory, the relationship was significant in Years 2 and 3 at both burned sites, while for the other groups of species, there were no consistent patterns (Table 2). The intercept of the regression decreased sharply from Year 1 to Year 2 in species with anemochory, moderately in species with zoochory and lightly in species with autochory (Table 2). Only in species with zoochory at the Valbermejo site, the intercept value did not decrease between Years 1 and 2. The turnover component of beta diversity showed significant relationships with difference in woody cover only for species with anemochory or zoochory, in Years 2 or 3, with no consistent patterns (Table 2). In the case of the nestedness component, this relationship was significant for species with anemochory in Year 3 at both burned sites and at the unburned site, while for species with zoochory, it was only significant in Year 1 at the Valbermejo site (Table 2).

## 4 DISCUSSION

Our results show a system with high beta diversity that is dominated by the turnover component both in the absence of fire and in the first three postfire years, implying a pattern of replacement of species identities rather than one of diversity hotspots. The nearly ubiquitous pattern of increasing community dissimilarity with spatial distance (i.e., distance decay), driven almost exclusively by the turnover component, together with the stronger spatial patterns of the group of species with less dispersal ability, suggests that the main driver of community assembly was dispersal limitation. Biotic interactions with woody plants contributed less to community dissimilarity, and this tended to occur more frequently through the nestedness component, suggesting some degree of competitive exclusion. Furthermore, the effect of biotic interactions with woody plants was more important in the group of species with anemochory, indicating that species with high dispersal ability were able to track suitable sites through dispersal.

### 4.1 Multiple‐site dissimilarities

Multiple‐site beta diversity remained extremely high, even if species richness changed up to almost twofold—either positive or negative—from year to year. This suggests an extremely diverse and dynamic community in the postfire environment (Viedma et al., 2012). The high beta diversity of Year 1 may be due to the effect of the high fire severity typical of shrubland fires, and that has been found to increase beta diversity in vegetation by enhancing small‐scale heterogeneity (Heydari et al., 2017). The decrease in beta diversity in Year 2, although minor, suggests a homogenization of the community caused by the increase in abundance of herbaceous species, as was shown by the marked increase in herbaceous species cover. This is a common pattern in fire‐prone Mediterranean ecosystems, where maximum species richness (alpha diversity) is found shortly after fire (Calvo et al., 2005; Keeley et al., 2005, 2012; Parra & Moreno, 2018; Pérez & Moreno, 1998). Furthermore, Keeley et al. (2005) found that, in California chaparral, species richness increased again the fifth year after fire, and explained it as being due to mass effects (Shmida & Wilson, 1985) rather than colonization, as most species were already present somewhere in the burned area the first postfire year. This effect could explain the decrease in beta diversity in Year 2, where species richness at the 1 m^2^ quadrats (alpha diversity) increased sharply while the increase in the total pool of species (gamma diversity) was moderate.

The main component of beta diversity was turnover, which is a dominant pattern of community variation across organisms and studies (Heydari et al., 2017; Si et al., 2015; Soininen et al., 2018; Vanneste et al., 2020), including after fire (Han et al., 2018). Surprisingly, the contribution of nestedness to total beta diversity was higher in species with the high dispersal ability (anemochory and zoochory) rather than in those with no dispersal traits (autochory), which is contrary to what broader‐scale studies have found for species with low dispersal ability (e.g., Dobrovolski et al., 2012; Hill et al., 2017; Si et al., 2015). However, this is not a universal pattern and, for instance, Aranda et al. (2013) found no differences in nestedness‐resultant dissimilarity between groups with contrasting dispersal abilities (bryophytes, pteridophytes, and seed plants) in Macaronesian plants. This pattern might be related to intrinsic organismal features, as Soininen et al. (2018) found in a meta‐analysis, where passively dispersed taxa (as in the case of anemochory or zoochory) had a very low turnover component and beta diversity.

The overall high values of beta diversity that we found are, however, highly related to sampling scale. At the fine‐grained spatial scales of our study, beta diversity tends to be high due to geometric reasons related to mean occupancy of species in samples (Storch, 2016). In a study in grassland plots of sizes comparable to those of our study, Dembicz et al. (2021) analyzed the z coefficient of the power law species‐area relationship, a parameter that is a measure of beta diversity (Koleff et al., 2003). They showed that factors that affect plant cover and/or number of individuals have direct effects on beta diversity by increasing or decreasing the number of subplots occupied by individual species, thus increasing or decreasing similarity in species composition. This helps explain the decrease in beta diversity in our burned sites the second year after fire, and especially, the great decrease in the intercept of the pairwise beta diversity relationships with spatial distance or with differences in woody cover in Year 2 (see Discussion below).

### 4.2 Pairwise dissimilarities and drivers of community assembly

#### 4.2.1 Spatial distance

We found a clear pattern of distance decay of similarity whereby samples further apart contained increasingly different species assemblages, suggesting an important role of dispersal limitation. This pattern was dominated by the turnover component, meaning that the differences between sites further apart were due to replacement of species identities, and not to some sites being poorer subsets of richer sites (i.e., not caused by nestedness). Distance decay in similarity is a universal pattern at regional and continental scales (Keil et al., 2012; Soininen, Mcdonald, & Hillebrand, 2007b), and turnover is the dominant component in different biological groups over such geographical extents (Keil et al., 2012; Soininen et al., 2018). Keil et al. (2012) interpreted this pattern as a sign that species distributions are not in equilibrium with current environmental conditions in Europe, and that dispersal limitation and historical processes are still shaping large‐scale species distributions. This may also be the case at the burned sites, where the postfire community could be out of equilibrium after the disturbance. This pattern was already present in Year 1, which may be due to several factors. First, prefire spatial patterns in community composition may have persisted with species that survived fire in the seed bank (Torres et al., 2013). An examination of aerial images from the mid‐20th century supports this hypothesis, with a marked gradient in vegetation structure being appreciated at the Valbermejo site (Torres, 2012). These patterns in seed bank may be modified by variation in fire intensity, which can filter the species that will finally emerge after fire (Harms et al., 2017; Heydari et al., 2017; Odion & Davis, 2000). Finally, new spatial patterns in community composition may be created by immigration of species from unburned sources (Rodrigo et al., 2012). It is likely that these processes contributed jointly to the observed patterns, and more importantly, both contributed to increase compositional differences, as Rodrigo et al. (2012) found that there were significant differences in species composition between inputs from the seed bank and from seed rain. The spatial patterns of dissimilarity remained all 3 years after fire and even increased in Year 3, being also present in the adjacent unburned stand. This strongly suggests that a hypothetical equilibrium has not yet been reached, and that distance decay is an intrinsic property of biological communities regardless of spatial scale. Rather, dispersal limitation is responsible for the high turnover observed even at such small spatial scales (Harms et al., 2017; Kraft & Ackerly, 2014). This conclusion is also consistent with the Carousel model by van der Maarel and Sykes (1993), in which species can move within the site at short distances by stochastic dispersal. Therefore, this might indicate that short‐distance seed dispersal plays a key role in the community assembly of both early postfire communities and unburned ones. This is supported by the fact that the intensity of the relationship with spatial distance (the slope of the regression) was higher in Year 3 in species with autochory, and much lower in species with anemochory. Similar results have been found in other high‐diversity ecosystems such as longleaf pine savannas (Harms et al., 2017; Myers & Harms, 2011), which are unsaturated in species and experience changes in biodiversity and species composition when dispersal increases.

The pairwise relationships of dissimilarity with distance also revealed an interesting pattern in the sharp drop of the intercept of the regression with spatial distance (i. e., estimated dissimilarity at a spatial distance of 0) from Year 1 to Year 2 after fire. Although this is a measure of pairwise dissimilarity, which does not reflect total heterogeneity in the pool of sampling quadrats (Baselga, 2013), it clearly points to a generalized homogenization of adjacent pairs of sites. A likely explanation is an increase in the abundance of individuals (indicated by the increase in herbaceous cover), which would increase the shared presences of species in adjacent pairs of plots (Dembicz et al., 2021; Storch, 2016). This applied to the full set of species and to the groups of species with different dispersal abilities, but the decrease in dissimilarity was much stronger in species with anemochory, suggesting that although these species have the potential to disperse long distances, short‐distance dispersal was dominant (Cousens et al., 2008; Plue & Hermy, 2012). Considering that our species were herbaceous and therefore of short height, the potential for long‐distance dispersal decreases strongly, as height is a key factor in dispersal potential (Nathan et al., 2011). In the case of species with autochory, dispersal limitation was even more marked, occurring in the immediate vicinity of mother plants, thus maintaining a higher dissimilarity.

#### 4.2.2 Effects of woody plant cover

The significant relationship between pairwise beta diversity and differences in woody plant cover at both burned sites in Year 3 suggests some role of non‐trophic biotic interactions with woody plants in community assembly. However, the response over time was inconsistent between sites, as was the relationship with the turnover and nestedness components. This might indicate that the effect of interactions with woody plants is less important for community assembly than that of dispersal limitation and spatial distance. In the cases of significant relationship with the turnover component, it might be due to niche processes taking place as the canopy closes, promoting the establishment of different species assemblages in the different microenvironments (Måren et al., 2018). On the contrary, the relationship with the nestedness component indicates a negative effect of woody plants, which exclude some species and create species‐poor sites that contrast with species‐rich assemblages in more open areas, although this was only observed at one of the burned sites. These results are in line with what has been found in postfire coastal heathlands in northern Europe, where niche‐driven dynamics become more important in late successional stages, associated with the development of vegetation cover (Måren et al., 2018). This effect is also found in mature stands not affected by fire, where the structural heterogeneity created by trees is an important driver of community assembly (Kouba et al., 2014; Sabatini et al., 2014). Han et al. (2018) found, in burned sites in southwest China, that environmental drivers were more important than spatial distance in community assembly after fire, but they sampled a wider range of environmental conditions including different topographical positions (hilltop to valley bottom).

We found no consistent relationships between woody plant cover and beta diversity or its components for the different groups of species, and the relationships were rather weak, suggesting that biotic interactions are not an important driver of community assembly in relation to dispersal ability. However, in the case of species with anemochory, the significant relationship of the nestedness component in Year 3 and at the unburned stand indicates that these species, with more mobility, can track suitable sites—open areas—more efficiently (Gianuca et al., 2017), creating richer subsets compared with the understory of woody plants, where a poorer subset of species would remain.

## 5 CONCLUSION

This work shows that local scale beta diversity is dominated by species turnover, where species identities change across neighboring locations, while the accumulation of species in diversity hotspots (i.e., nestedness) is a minor component of beta diversity. Furthermore, we show that spatial distance explains better composition dissimilarity, likely an indicator of the role of dispersal limitation in local community assembly during postfire succession, while interactions with woody plants are a less important contributor to community assembly. This work helps understand fine‐scale community assembly mechanisms in highly dynamic, postfire communities.

## AUTHOR CONTRIBUTIONS

**Iván Torres Galán:** Conceptualization (equal); data curation (equal); formal analysis (equal); investigation (equal); writing – original draft (lead); writing – review and editing (equal). **Antonio Parra:** Formal analysis (equal); investigation (equal); writing – review and editing (equal). **José M Moreno:** Conceptualization (equal); funding acquisition (lead); resources (lead); writing – review and editing (equal).

## CONFLICT OF INTEREST

None declared.

## Supporting information


Appendix S1

## Data Availability

The data that support the findings of this study are openly available in Dryad at: https://doi.org/10.5061/dryad.7h44j0zx5.
