# Supplementary material for: Effects of spatial distance and woody plant cover on beta diversity point to dispersal limitation as a driver of community assembly during postfire succession in a Mediterranean shrubland
Source: Ecol Evol. 2022 Jul 24;12(7):e9130. doi: 10.1002/ece3.9130 (PMC9309027; doi:10.1002/ece3.9130)
Supplement: Supplementary file 1 — Appendix S1 [file ECE3-12-e9130-s001.docx]

**Appendix 1.** List of species names, abbreviated name and dispersal mode assigned, of herbaceous vascular plants in the first three years after a fire in a Mediterranean shrubland and in an adjacent unburned stand. Nomenclature follows Devesa (1995).

| **Species name** | **Abbreviation** | **Dispersal mode** |
| --- | --- | --- |
| *Agrostis castellana* Boiss. & Reut. | Agr.cas | Zoochory |
| *Agrostis nebulosa* Boiss. & Reut. | Agr.neb | Autochory |
| *Agrostis pourretii* Willd. | Agr.pou | Zoochory |
| *Aira caryophyllea* L. | Air.car | Zoochory |
| *Aira cupaniana* Guss. | Air.cup | Zoochory |
| *Airopsis tenella* (Cav.) Coss. & Durieu | Air.ten | Autochory |
| *Anagallis arvensis* L. | Ana.arv | Autochory |
| *Anagallis monelli* L. | Ana.mon | Autochory |
| *Anarrhinum bellidifolium* (L.) Willd. | Ana.bel | Autochory |
| *Andryala integrifolia* L. | And.int | Anemochory |
| *Andryala laxiflora* DC. | And.lax | Anemochory |
| *Anemone palmata* L. | Ane.pal | Autochory |
| *Anthemis arvensis* L. | Ant.arv | Autochory |
| *Anthoxanthum aristatum* Boiss. | Ant.ari | Zoochory |
| *Aphanes cornucopioides* Lag. | Aph.cor | Autochory |
| *Arabidopsis thaliana* (L.) Heynh. in Holl & Heynh. | Ara.tha | Autochory |
| *Aristolochia pistolochia* L. | Ari.pis | Autochory |
| *Arrhenatherum elatius* (L.) J. Presl & C. Presl | Arr.ela | Zoochory |
| *Aster squamatus* (Spreng.) Hieron. | Ast.squ | Anemochory |
| *Asterolinon linum-stellatum* (L.) Duby in DC. | Ast.lin | Autochory |
| *Avellinia michelii* (Savi) Parl. | Ave.mic | Zoochory |
| *Avena barbata* Link | Ave.bar | Zoochory |
| *Avena sterilis* L. | Ave.ste | Zoochory |
| *Bellardia trixago* (L.) All. | Bel.tri | Autochory |
| *Brachypodium distachyon* (L.) P. Beauv. | Bra.dis | Zoochory |
| *Briza maxima* L. | Bri.max | Anemochory |
| *Briza minor* L. | Bri.min | Anemochory |
| *Bromus hordeaceus* L. | Bro.hor | Zoochory |
| *Bromus matritensis* L. | Bro.mat | Zoochory |
| *Campanula lusitanica* L. | Cam.lus | Autochory |
| *Campanula rapunculus* L. | Cam.rap | Autochory |
| *Cardamine hirsuta* L. | Car.hir | Autochory |
| *Carduus tenuiflorus* Curtis | Car.ten | Anemochory |
| *Carlina corymbosa* L. | Car.cor | Anemochory |
| *Centaurea melitensis* L. | Cen.mel | Autochory |
| *Centaurium erythraea* Rafn | Cen.ery | Autochory |
| *Centaurium maritimum* (L.) Fritsch | Cen.mar | Autochory |
| *Centranthus calcitrapae* (L.) Dufr. | Cen.cal | Anemochory |
| *Cephalanthera longifolia* (L.) Fritsch | Cep.lon | Anemochory |
| *Cerastium glomeratum* Thuill. | Cer.glo | Autochory |
| *Chaetonychia cimosa* (L.) Sweet | Cha.cim | Autochory |
| *Chaetopogon fasciculatus* (Link) Hayek | Cha.fas | Zoochory |
| *Chamaemelum mixtum* (L.) All. | Cha.mix | Autochory |
| *Chenopodium botrys* L. | Che.bot | Autochory |
| *Chondrilla juncea* L. | Cho.jun | Anemochory |
| *Coleostephus myconis* (L.) Rchb. | Col.myc | Autochory |
| *Conyza canadensis* (L.) Cronq. | Con.can | Anemochory |
| *Coronilla dura* (Cav.) Boiss. | Cor.dur | Autochory |
| *Crassula tillaea* Lest.–Garl. | Cra.til | Autochory |
| *Crepis capillaris* (L.) Wallr. | Cre.cap | Anemochory |
| *Crepis foetida* L. | Cre.foe | Anemochory |
| *Crepis vesicaria* L. | Cre.ves | Anemochory |
| *Chrozophora tinctoria* (L.) Raf. | Chr.tin | Autochory |
| *Crucianella angustifolia* L. | Cru.ang | Autochory |
| *Cuscuta epithymum* (L.) L. | Cus.epi | Autochory |
| *Cynosurus echinatus* L. | Cyn.ech | Zoochory |
| *Dactylis glomerata* L. | Dac.glo | Zoochory |
| *Daucus carota* L. | Dau.car | Zoochory |
| *Daucus durieua* Lange | Dau.dur | Zoochory |
| *Delphinium gracile* DC. | Del.gra | Autochory |
| *Dipcadi serotinum* (L.) Medik. | Dip.ser | Autochory |
| *Echium plantagineum* L. | Ech.pla | Autochory |
| *Eryngium tenue* Lam. | Ery.ten | Autochory |
| *Euphorbia exigua* L. | Eup.exi | Zoochory |
| *Euphorbia falcata* L. | Eup.fal | Zoochory |
| *Evax carpetana* Lange | Eva.car | Autochory |
| *Filago lutescens* Jord. | Fil.lut | Anemochory |
| *Fumaria* sp. | Fum.sp | Autochory |
| *Galactites tomentosa* Moench | Gal.tom | Anemochory |
| *Galium parisiense* L. | Gal.par | Zoochory |
| *Gastridium ventricosum* (Gouan) Schinz & Thell. | Gas.ven | Zoochory |
| *Gaudinia fragilis* (L.) P. Beauv | Gau.fra | Zoochory |
| *Geranium robertianum* L. | Ger.rob | Zoochory |
| *Gladiolus communis* L. | Gla.com | Anemochory |
| *Gnaphalium luteo-album* L. | Gna.lut | Anemochory |
| *Heliotropium europaeum* L. | Hel.eur | Autochory |
| *Herniaria lusitanica* Chaudhri | Her.lus | Autochory |
| *Holcus annuus* C. A. Mey. | Hol.ann | Zoochory |
| *Holcus lanatus* L. | Hol.lan | Zoochory |
| *Hymenocarpos hispanicus* Lassen | Hym.lot | Autochory |
| *Hypericum perforatum* L. | Hyp.per | Autochory |
| *Hypochaeris glabra* L. | Hyp.gla | Anemochory |
| *Hypochaeris radicata* L. | Hyp.rad | Anemochory |
| *Jasione montana* L. | Jas.mon | Autochory |
| *Juncus bufonius* L. | Jun.buf | Autochory |
| *Juncus capitatus* Weigel | Jun.cap | Autochory |
| *Lactuca serriola* L. | Lac.ser | Anemochory |
| *Lactuca viminea* (L.) J. & C. Presl | Lac.vim | Anemochory |
| *Lamarckia aurea* (L.) Moench | Lam.aur | Zoochory |
| *Lathyrus angulatus* L. | Lat.ang | Autochory |
| *Leontodon taraxacoides* (Vill.) Mérat | Leo.tar | Anemochory |
| *Linaria spartea* (L.) Chaz. | Lin.spa | Autochory |
| *Linum trigynum* L. | Lin.tri | Autochory |
| *Logfia gallica* (L.) Coss. & Germ. | Log.gal | Anemochory |
| *Logfia minima* (Sm.) Dumort. | Log.min | Anemochory |
| *Lolium rigidum* Gaudin | Lol.rig | Autochory |
| *Lotus conimbricensis* Brot. | Lot.con | Autochory |
| *Lotus parviflorus* Desf. | Lot.par | Autochory |
| *Malva hispanica* L. | Mal.his | Autochory |
| *Melica magnolii* Gren. & Godr. | Mel.mag | Anemochory |
| *Mercurialis ambigua* L. fil. | Mer.amb | Autochory |
| *Micropyrum tenellum* (L.) Link | Mic.ten | Zoochory |
| *Misopates orontium* (L.) Raf. | Mis.oro | Autochory |
| *Molineriella laevis* (Brot.) Rouy | Mol.lae | Zoochory |
| *Myosotis discolor* Pers. | Myo.dis | Autochory |
| *Narcissus triandrus* L. | Nar.tri | Autochory |
| *Ornithogalum umbellatum* L. | Orn.umb | Autochory |
| *Ornithopus compressus* L. | Orn.com | Autochory |
| *Orobanche ramosa* L. | Oro.ram | Autochory |
| *Ortegia hispanica* Loefl. ex L. | Ort.his | Autochory |
| *Papaver pinnatifidum* Moris | Pap.pin | Autochory |
| *Parentucellia viscosa* (L.) Caruel | Par.vis | Autochory |
| *Paronychia argentea* Lam. | Par.arg | Autochory |
| *Paronychia echinulata* A. O. Chater | Par.ech | Autochory |
| *Petrorrhagia nanteuilii* (Burnat) P. W. Ball & Heywood | Pet.nan | Autochory |
| *Phalaris minor* Retz. | Pha.min | Autochory |
| *Plantago coronopus* L. | Pla.cor | Autochory |
| *Plantago lanceolata* L. | Pla.lan | Autochory |
| *Platycapnos spicata* (L.) Bernh. | Pla.spi | Autochory |
| *Polycarpon tetraphyllum* (L.) L. | Pol.tet | Autochory |
| *Polypogon maritimus* Willd. | Pol.mar | Zoochory |
| *Pseudoscabiosa diandra* (Lag.) Greuter & Burdet | Pse.dia | Anemochory |
| *Psilurus incurvus* (Gouan) Schinz & Thell. | Psi.inc | Zoochory |
| *Pulicaria paludosa* Link | Pul.pal | Anemochory |
| *Raphanus raphanistrum* L. | Rap.rap | Autochory |
| *Rubia peregrina* L. | Rub.per | Zoochory |
| *Rumex angiocarpus* Murb. | Rum.ang | Autochory |
| *Rumex bucephalophorus* L. | Rum.buc | Zoochory |
| *Sagina apetala* Ard. | Sag.ape | Autochory |
| *Sanguisorba verrucosa* (G. Don) Ces. | San.ver | Autochory |
| *Senecio jacobaea* L. | Sen.jac | Anemochory |
| *Senecio lividus* L. | Sen.liv | Anemochory |
| *Sesamoides purpurascens* (L.) G.López | Ses.pur | Autochory |
| *Sherardia arvensis* L. | She.arv | Autochory |
| *Silene gallica* L. | Sil.gal | Autochory |
| *Silene inaperta* L. | Sil.ina | Autochory |
| *Solanum nigrum* L. | Sol.nig | Zoochory |
| *Sonchus oleraceus* L. | Son.ole | Anemochory |
| *Spergula arvensis* L. | Spe.arv | Autochory |
| *Spergula pentandra* L. | Spe.pen | Anemochory |
| *Spergularia rubra* (L.) J. Presl & C. Presl | Spe.rub | Autochory |
| *Stachys arvensis* (L.) L. | Sta.arv | Autochory |
| *Taeniatherum caput-medusae* (L.) Nevski | Tae.cap | Zoochory |
| *Teesdalia coronopifolia* (J. P. Bergeret) Thell. | Tee.cor | Autochory |
| *Thapsia villosa* L. | Tha.vil | Anemochory |
| *Tolpis barbata* (L.) Gaertn. | Tol.bar | Anemochory |
| *Tolpis umbellata* Bertol. | Tol.umb | Anemochory |
| *Trifolium angustifolium* L. | Tri.ang | Zoochory |
| *Trifolium arvense* L. | Tri.arv | Zoochory |
| *Trifolium bocconei* Savi | Tri.boc | Autochory |
| *Trifolium campestre* Schreb. | Tri.cam | Autochory |
| *Trifolium cherleri* L. | Tri.che | Zoochory |
| *Trifolium gemellum* Willd. | Tri.gem | Zoochory |
| *Trifolium glomeratum* L. | Tri.glo | Autochory |
| *Trifolium* sp. | Tri.sp | NA |
| *Trifolium striatum* L. | Tri.str | Zoochory |
| *Xolantha guttata* (L.) Raf. | Tub.gut | Autochory |
| *Veronica arvensis* L. | Ver.arv | Autochory |
| *Vicia disperma* DC. | Vic.dis | Autochory |
| *Viola arvensis* Murray | Vio.arv | Zoochory |
| *Vulpia ciliata* Dumort. | Vul.cil | Zoochory |
| *Vulpia myuros* (L.) C. C. Gmel. | Vul.myu | Zoochory |

Devesa, J.A. (1995). *Vegetación y Flora de Extremadura*. Universitas, Badajoz.
